# The fight against COVID-19 in sub-Saharan Africa-a threat to the continuous management of HIV patients: application of the action areas of the Ottawa charter for health promotion

**DOI:** 10.11604/pamj.supp.2020.35.2.23224

**Published:** 2020-05-07

**Authors:** Elvis Enowbeyang Tarkang

**Affiliations:** 1 Department of Population and Behevioural Sciences, School of Public Health, University of Health and Allied Sciences, Ho, Volta Region, Ghana; 2 HIV/AIDS Prevention Research Network Cameroon, Kumba, Southwest Region, Cameroon

**Keywords:** Sub-Saharan Africa, COVID-19, HIV management, Ottawa charter for health promotion, antiretroviral therapy

## Abstract

The Novel Coronavirus (2019-nCoV) was detected in December 2019 in the Hubei Province of China. Also known as COVID-19, the outbreak was declared a pandemic by the World Health Organization (WHO) in March 2020. Sub-Saharan Africa (SSA), which is the region hardest hit by HIV, is also highly affected by COVID-19. The fight against COVID-19 in SSA might threaten the continuous management of persons living with HIV (PLHIV). This commentary uses the five action areas of the Ottawa charter for health promotion to address the issue. If the issues raised in this commentary are not addressed quickly by SSA governments, every single link of the supply chain in the HIV response will be disrupted. This disruption might result in more stock-outs, shortages and a lack of access to ART in the months ahead. The SSA governments must ensure that HIV treatment adherence is not compromised owing to a shift of focus to the fight against COVID-19. They should ensure that everyone on HIV treatment gets an adequate supply of antiretroviral therapy (ART).

## Commentary

On December 31, 2019, there was an outbreak of a respiratory illness in Wuhan, Hubei Province, China [1]. By January 7, 2020, the illness was identified as a Novel Coronavirus (2019-nCoV). Coronaviruses constitute a huge family of viruses that cause upper-respiratory-tract infections. COVID-19 was declared a global pandemic by the WHO on March 11, 2020 [2]. Like a pandemic, 213 countries, areas or territories [3], out of the 241 recognised by the WHO had recorded COVID-19 cases as of April 25, 2020 [4]. To date, a total of 2,686,785 confirmed cases with 184,681 deaths were recorded worldwide. In sub-Saharan Africa (SSA), 18,731 cases were recorded with 808 deaths [3]. Globally, 37.9 million people are living with HIV with 1.7 million newly infected cases. Sub-Saharan Africa (SSA) is the most severely affected region with nearly one in every twenty-five adults (3.9%) living with HIV and this accounts for two thirds (25.7 million) of all persons living with HIV (PLHIV) globally. This sub-region also accounts for about 70% of new infections and 74% of HIV-related deaths [5]. Therefore, PLHIV in SSA should be on continuous management in the midst of the COVID-19 pandemic.

### Facts about COVID-19 and Human Immunodeficiency Virus

Before the declaration of COVID-19 as a pandemic, the world was focused on combatting HIV and its comorbidities. The weak immune status that makes PLHIV at an increased risk of contracting opportunistic infections like Tuberculosis, also makes them vulnerable to COVID-19 [6]. However, currently, there is no empirical evidence linking PLHIV who are adhering to antiretroviral therapy (ART) to a higher risk of contracting COVID-19. People living with HIV who have a compromised immune system because they are not adhering to ART should be extra cautious to prevent COVID-19 infection. This is because their immune system may not be strong enough to deal with the virus. Also, PLHIV are more vulnerable to respiratory infections if they do not adhere to treatment. Therefore, it is very important that patients adhere to their ART as prescribed, during this period of COVID-19, since COVID-19 also causes upper respiratory tract infections.e process of getting ART to PLHIV. Therefore, task-shifting of HIV care to communities will be crucial during this pandemic, such as allowing for supervised community distribution of ART. The WHO preventive measures against COVID-19 in the general population also apply to PLHIV. These include: washing hands frequently with soap and water for at least 40 seconds, using an alcohol-based hand sanitiser for situations where there is no access to soap and water, avoid touching face because this is one of the ways the virus enters the body, maintaining social distancing of at least 1 metre, covering nose and mouth with a clean tissue when sneezing or coughing and throwing the tissue away and washing hands, and in the absence of a tissue, using the inside of the elbow to cover the mouth and nose [7]. Following the WHO recommendations [7], governments in SSA have implemented several measures to curb the COVID-19 pandemic such as total lockdown and restricting movements, among others. This fight against COVID-19 could impact negatively on the HIV response in SSA. Because of the increasing number of positive cases for COVID-19, the already weak health systems in SSA countries are being stretched and there are increased demands for more resources to fight the pandemic to the detriment of other services and sectors, including HIV services.

As cases of COVID-19 keep rising in SSA, so are concerns about the impact of the pandemic on the HIV response and PLHIV. If the pandemic is not arrested quickly in SSA countries which are already burdened with HIV, it will have significant implications for the continuous management of PLHIV as much of the resources are now channelled to combat COVID-19 to the detriment of the HIV response. It is imperative that the COVID-19 response in SSA does not affect the continuity of services for PLHIV because funds for HIV response may be depleted as a result of unexpected expenses in combatting COVID-19 such as procurement of sanitisers and PPE for field staff, the treatment and management of COVID-19 patients, among others. As health systems become more overwhelmed with the need to treat COVID-19 patients, other services such as HIV services may get deprioritized. The consequence is that PLHIV who require access to ART to stay healthy may find it difficult to adhere to treatment. This is coupled with concerns that lockdowns imposed by countries in SSA to curb COVID-19 could prevent PLHIV from leaving their homes to access their medications, while health workers in the HIV response could be infected with COVID-19 or be unwilling to continue working without PPE. Therefore, the COVID-19 pandemic could disrupt access to treatment, testing and supply chains for people affected by HIV. The SSA governments should, therefore, take proactive measures to prevent this from happening.

**Application of the Ottawa Charter for Health Promotion in addressing the issue:** health promotion was defined as the process of enabling people to increase control over and to improve their physical, mental and social wellbeing [8]. There are five key action areas of the Ottawa Charter for Health Promotion, which provide strategies from which governments in SSA can support the continuity of HIV services amidst the COVID-19 pandemic. These are building healthy public policies, creating a supportive environment, strengthening community action, developing personal skills and re-orienting health services [8]. For any intervention aimed at the continuous management of PLHIV amidst COVID-19 in SSA to be successful, it should target these five action areas:

**Building healthy public policies:** healthy policies include legislation, fiscal measures, taxation and organizational change. It aims to foster greater equity and to make the healthier choice the easier choice for the population and policy-makers as well [8]. Healthy public policies for the continuous management of PLHIV in SSA amidst COVID-19 include providing their rights and protection and the continuous provision of services to them. As the COVID-19 cases keep increasing and the number of patients in need of intensive care also increasing in SSA, PLHIV might no longer be able or willing to access health facilities to collect their medications. The governments should come out with a policy to integrate the COVID-19 prevention practices into HIV programming. In this regard, the governments should provide HIV centres with enough stock, so that they can rapidly scale up a multi-month (between 3 and 6 months) dispensing of ART and other commodities for PLHIV so that they can abide by the confinement measure adopted by countries to prevent COVID-19. This will prevent PLHIV from running out of medication should travel restrictions continue and would reduce the need for them to access the health services regularly, thereby reducing their risk of contracting the COVID-19 infection [9]. The governments should establish procedures and guidelines to track all PLHIV who are infected with COVID-19 and who may be admitted to hospital or quarantined at home to ensure that they maintain access to ART. This will ensure that they continue to adhere to their treatment amidst COVID-19. In the same vein, the governments should develop a policy to support a sustained delivery of HIV services by integrating social distancing measures in line with local and national efforts. This includes connection with PLHIV virtually and offering convenient long-term dispensing, pick-up and delivery of HIV commodities, services and medications [9]. Concerning HIV counselling and testing, the governments should develop policies to expand options for HIV testing that will reduce physical contacts such as HIV self-testing, facility pick-up, at-home testing and home delivery. There should be a policy that restricts ART clinic staff from being assigned to direct COVID-19 patient care and response, in order to avoid exposure, infection and possible quarantine of the entire ART clinic staff, which will eventually break the HIV service delivery chain [9].

**Creating a supportive environment:** a supportive environment is essential for health [10]. Supportive environments cover the physical, social, economic and political environment. Supportive environments encompass where people live, work and play [8]. Supportive environments are ones that provide PLHIV protection from threats to their health, resilience and overall development. A supportive environment for the continuous management of PLHIV amidst COVID-19 includes interventions to minimize stigma and discrimination and provide reliable access to relevant health and support services. Health personnel in charge of service delivery to PLHIV should be supported so that they can continue providing their services, by providing them with incentives and PPE to prevent COVID-19 among them and their patients. The governments should also ensure an adequate supply of running water and soap for handwashing, alcohol-based hand sanitisers, face masks, thermometers and other PPE at the HIV facility and community levels to mitigate the effect of COVID-19 on the HIV response [9]. The government should provide a protocol on how to respond to PLHIV if they or their recent contacts experience COVID-19 symptoms or test positive. HIV patients with COVID-19 symptoms should be supported not to come for ART, but should rather be referred for screening while sending their relatives to come for the drug on their behalf.

**Strengthening community action:** effective health promotion strategies involve empowering the communities, the ownership and control of their own endeavours and destinies [8]. Strengthening community action for the continuous management of PLHIV amidst COVID-19 aims to provide direct support to them and also support Non-Governmental Organisations (NGOs) and Community-Based Organisations (CBOs) working with PLHIV. The circumstances around the COVID-19 pandemic may complicate efforts by NGOs and CBOs aimed at identifying new HIV cases, such as outreach activities and recruiting them to come to health facilities to get tested and subsequently be placed on ART. The HIV response in SSA relies heavily on NGOs, CBOs and peer support groups who monitor and follow up PLHIV in the communities. Due to the social isolation imposed by most countries in SSA to curb COVID-19, it is now difficult for these organisations in the HIV service delivery chain to function effectively. These groups work in the communities to make sure that PLHIV are sound and that they have the drugs they need and are adhering to them. The governments should, therefore, support the activities of NGOs and CBOs concerned with the HIV response with android phones and airtime so that they can continue to monitor PLHIV via mobile platforms [9].

**Developing personal skills:** health promotion supports personal and social development through providing information, education for health and enhancing life skills. By so doing, it increases the options available to PLHIV to exercise more control over their own health and over their environments and to make choices conducive to health [8]. All staff providing HIV services should be trained on COVID-19 transmission, symptoms, prevention and implication for PLHIV so that they will be knowledgeable in mitigating the effect of the pandemic on PLHIV. There is also the need for continuous education at community levels to enable PLHIV to proactively go to get their ART supplies before health facilities get overwhelmed with COVID-19 patients. The SSA governments should as a matter of urgency regularly provide reliable information on COVID-19 and HIV to PLHIV via text messages so that they can be well equipped to prevent the pandemic [9].

**Reorienting the health services:** according to the Ottawa Charter, the responsibility for health promotion in health services is shared among individuals, community groups, health professionals, health service institutions and governments [8]. The role of the health sector must, therefore, move increasingly in a health promotion direction, beyond its primary responsibility of providing clinical and curative services. Supply chains for ART in most SSA countries revolve around one-month dispensing, whereas in others there are limited supplies of drugs. Most governments have only approved one-month dispensing and patient visits are scheduled around one-month intervals. This is not practicable in the midst of confinement and social distancing imposed by countries to combat COVID-19. The SSA governments should reorient the HIV services to provide at least three months (and ideally six months) of ART to PLHIV in response to COVID-19. Health professionals can then be empowered to use phone calls or electronic follow-up to support adherence to drugs and to give guidance on how to manage side effects [9]. Furthermore, the impact of COVID-19 on supply chains might, in turn, affect the process of getting ART to PLHIV. Therefore, task-shifting of HIV care to communities will be crucial during this pandemic, such as allowing for supervised community distribution of ART.

## Conclusion

If the issues raised in this commentary are not addressed quickly by SSA governments, every single link of the supply chain in the HIV response will be disrupted. This disruption is going to result in more stock-outs, shortages and a lack of access to ART in the months ahead. As a result, HIV patients in SSA will not be able to adhere to their treatment plan, leading to a compromised immune system and an increased in viral load, thus putting the patients at risk of opportunistic infections, including COVID-19. The SSA governments must ensure that HIV treatment adherence is not compromised owing to a shift of focus to the fight against COVID-19. They should ensure that everyone on HIV treatment gets an adequate supply of ART.
